# A Novel Xenogeneic Co-Culture System to Examine Neuronal Differentiation Capability of Various Adult Human Stem Cells

**DOI:** 10.1371/journal.pone.0024944

**Published:** 2011-09-14

**Authors:** Anna E. Petschnik, Benjamin Fell, Stephan Tiede, Jens K. Habermann, Ralph Pries, Charli Kruse, Sandra Danner

**Affiliations:** 1 Fraunhofer Research Institution for Marine Biotechnology, Lübeck, Germany; 2 Department of Dermatology, Allergology and Venerology, University of Lübeck, Lübeck, Germany; 3 Department of Surgery, University of Lübeck, Lübeck, Germany; 4 ENT Department, University of Lübeck, Lübeck, Germany; City of Hope National Medical Center and Beckman Research Institute, United States of America

## Abstract

**Background:**

Targeted differentiation of stem cells is mainly achieved by the sequential administration of defined growth factors and cytokines, although these approaches are quite artificial, cost-intensive and time-consuming. We now present a simple xenogeneic rat brain co-culture system which supports neuronal differentiation of adult human stem cells under more *in vivo*-like conditions.

**Methods and Findings:**

This system was applied to well-characterized stem cell populations isolated from human skin, parotid gland and pancreas. In addition to general multi-lineage differentiation potential, these cells tend to differentiate spontaneously into neuronal cell types *in vitro* and are thus ideal candidates for the introduced co-culture system. Consequently, after two days of co-culture up to 12% of the cells showed neuronal morphology and expressed corresponding markers on the mRNA and protein level. Additionally, growth factors with the ability to induce neuronal differentiation in stem cells could be found in the media supernatants of the co-cultures.

**Conclusions:**

The co-culture system described here is suitable for testing neuronal differentiation capability of numerous types of stem cells. Especially in the case of human cells, it may be of clinical relevance for future cell-based therapeutic applications.

## Introduction

The prevalence of neurodegenerative disorders, brain and spinal cord injury as well as stroke is furthermore increasing. Apart from medical treatment that can partially relieve symptoms there has been only little progress on regenerative medical therapy approaches of these diseases. In this regard, a cell-replacement therapy can be a promising approach. There is a promising effort to generate neuron-like cells from embryonic stem (ES) cells [1], [2], [3]. Therefore, despite the remarkable potential of ES cells, the obvious limit for a cell replacement therapy is the need for a match in respect to the major histocompatibility complex I [4]. Moreover, the use of human ES cells bears beside multiple ethical problems [5], the necessity of advanced cultivation techniques (e.g. feeder layer) [6], as well as the risk of tumorigenicity [7]. To avoid these problems, the use of autologous adult stem cells (SCs) would be an appropriate alternative.

Several studies have shown that human adult SCs from the bone marrow are capable of differentiating into neuron-like cells under specific conditions *in vitro* [8], [9], [10]. In particular, the defined application of soluble factors is known to stimulate neuronal differentiation. Such factors include retinoic acid [11], nerve growth factor [12], fibroblast growth factor [13] and epidermal growth factor [14]. However, all of these methods have certain features in common: they are cost- and labor-intensive as well as artificial and limited in their use of single factors. In regard to the intention of differentiating SCs in the course of autologous transplantation therapies, it would therefore be advisable to establish a differentiation model similar to post-transplantational effects *in vivo*. Hereby, we investigated an *in vitro*, but clinically relevant neuronal differentiation model of stem cells from multiple sources. A recent work reports the successful use of a co-culture system with slices of rat brain striatum to induce neuronal differentiation in a neural SC line isolated from human fetal brain [15]. This approach indicates the possibility to benefit from the whole set of factors secreted by specific biopsies. However, until now no one has tested the potential of brain biopsies on neuronal differentiation of human adult SCs.

Therefore, we developed an attractive simple and yet efficient xenogeneic co-culture model with unsliced adult rat brain biopsies providing all required factors to activate neuronal differentiation pathways in adult human SCs. Brain biopsies and SCs were cultured in the same environment separated only by a porous membrane. This co-culture system could also be used to predict the signal crosstalk of stem cells and brain tissue as it mimics parts of the graft-host interaction *in vivo*.

To test if this system is useful to enrich neuronal differentiation capability of human adult SCs, we applied it on different adult SC populations derived from human skin and glandular tissues. Several glandular tissues have been shown to be a source of SCs with spontaneous multi-lineage differentiation potential. These glandular SC populations were generated from human pancreas (pancreatic stem cells, PSC) [16], parotid gland (parotid-derived stem cells, PDSC) [17] and sweat glands [18]. SC populations with similar properties were also isolated from adult human full skin biopsies (skin-derived stem cells, SDSC) [19]. Regarding their regeneration potential *in vivo* pancreatic and parotid SCs isolated from mouse and rat have been shown to accelerate wound healing in an animal model for dermal skin regeneration [20]. In addition, human SCs from skin and glandular tissues exhibit the ability to partially differentiate also into neuronal cells [19], [21]. Thus, these SC populations are suitable candidates to test the applicability of the proposed co-culture model.

Our overall aim was to establish a simple, time- and money-saving, but still effective method to analyze the differentiation capability of adult human SC populations into neuronal cell lineages under *in vivo*-like conditions. Therefore, we characterized and compared human SDSC with glandular SC populations from pancreas and parotid gland, by testing their neuronal-differentiation potential by co-culturing them with adult rat brain biopsies. The data described in the current study suggests that this approach may be widely applicable to many human stem cell populations.

## Methods

### Ethics Statement

All experiments were performed according to Helsinki guidelines, in compliance with national regulations for the experimental use of human material. Utilization of human biopsies for research purposes was approved by the ethics committee of the University Lübeck (reference number: 10–058). All patients gave written informed consent.

The isolation of brain biopsies from Sprague-Dawley rats was approved by the animal protection committee of the University Lübeck (reference number: 11/A07/09).

### Stem Cell Isolation

A biopsy of a human pancreas was obtained from a 42-year-old male patient with pancreatitis. For isolation of skin-derived SCs retroauricular adult uninflamed scalp skin was obtained from a 61-year-old female patient undergoing routine facelift surgery. Salivary gland tissue arose from a female patient at the age of 44.

Human SCs from these tissues were isolated as previously described [16]. First, pancreatic, parotid and skin tissues (each 2 cm^3^) were freed of adhering fat tissue and chopped into small pieces with surgical scissors. Shortly after, the tissues were treated twice (20 and 15 min) with digestion medium containing HEPES-Eagle medium (pH 7.4), 10 mM HEPES buffer, 70% (v/v) modified Eaglés medium, 0.5% (v/v) Trasylol (Bayer AG, Germany), 1% (w/v) bovine serum albumin, 2.4 mM CaCl_2_ and collagenase (0.63 PZ/mg, Serva, Germany) at 37°C. Prior to each digestion, the preparation mixture was aerated with a mixture of oxygen and carbon dioxide (95% (v/v)). In between the two digestion steps, the remaining tissue pieces were washed with isolation medium, being equal to digestion medium but lacking collagenase and mechanically minced again using small chirurgic scissors. After the second digestion step, the remaining tissue fragments were dissociated into even smaller pieces by up-and-down suction through different glass pipettes with progressively more restrictive orifices (10, 5 and 2 mL pipettes) and filtered through a nylon mesh (250 µm mesh). After centrifugation (850 rpm, 5 min) and further purification by washing in Dulbecco's modified Eagle's medium (DMEM, Gibco, Germany) supplemented with 20% fetal bovine serum (FBS), 1 U/mL penicillin and 10 mg/mL streptomycin (all PAA Laboratories, Austria), the cells were seeded into 25 cm^2^ flasks.

### Propagation of Stem Cells

After reaching confluence, cells were subcultured by treatment with 0.1% trypsin (PAA Laboratories, Austria) and reseeded in DMEM supplemented with 10% FBS, 1 U/mL penicillin and 10 mg/mL streptomycin (10% FCS-DMEM). Medium was changed every 3-4 days. In this study, cells from passage 8–11 were used.

### Co-culture Experiments

Two days prior to co-culture experiments, human adult SCs were seeded in 10% FCS-DMEM on 6 well culture plates (50,000 cells/ mL) containing uncoated glass cover slips for immunocytochemical analysis. Brain biopsies were obtained from 8 weeks old, male Sprague-Dawley rats. During the indirect co-culture experiment rat brain tissue (two 0.125 cm^3^ pieces of the cerebrum and two 0.125 cm^3^ pieces of the cerebellum) was separated from the SCs by tissue culture inserts (0.4 µm pore size; Greiner Bio-One). As controls, adult SC were cultured in 10% FCS-DMEM without the addition of brain biopsies. After 48 h of incubation, brain biopsies were removed and human SCs were prepared for further analysis. Co-culture experiments were done at least twice for each approach.

### RNA Isolation and qPCR

RNA was isolated from cells grown in 6-well culture plates. Total RNA isolation was performed using the RNeasy Plus Mini kit and the QIAcube (both Qiagen, Germany) for automated RNA isolation according to manufactureŕs protocols. RNA concentration was determined by measuring the absorbance at 260 nm applying a Nanodrop spectrophotometer (Peqlab, Germany). cDNA was synthesized from 1 µg total RNA using the QuantiTect reverse transcription kit including a gDNA digestion step. qPCR was carried out with 1 µL cDNA in a 25 µL reaction volume using the QuantiFast SYBR Green PCR kit and commercially available ready-to-use QuantiTect Primers (all Qiagen): *Cluster of Differentiation Antigen 9* (*CD9*, 95 bp), *Cytokeratin 18* (*CK18*, 97 bp), *c-Myc* (129 bp), *Ki67* (86 bp), *Krüppel-like Factor 4* (*Klf4*, 72 bp), *Myocyte-specific Enhancer Factor 2D* (*MEF2D*, 85 bp), *Nestin* (77 bp), *Neurofilament heavy chain* (*NF(H)*, 97 bp), *light chain* (*NF(L),* 99 bp) and *medium chain* (*NF(M)*, 74 bp), *Neuron-specific Enolase* (*NSE*, 61 bp), *Octamer-binding Transcription Factor 4* (*Oct4*, 75 bp), *Protein Gene Product 9.5* (*PGP9.5*, 134 bp), *Peroxisome Proliferator-activated Receptor gamma* (*PPARγ,* 113 bp), *S100β* (64 bp), *SRY (Sex determining Region Y)-Box 2* (*Sox2*, 64 bp), *Secreted Phosphoprotein 1* (*SPP1,* 115 bp), *von-Willebrand-Factor* (*vWF*, 108 bp), *alpha-Smooth Muscle Actin* (α*-SMA*, 83 bp), *β-Actin* (146 bp), *β III Tubulin* (78 bp). Real-time quantifications were performed in duplicates using the Mastercycler ep realplex (Eppendorf, Germany). The amplification cycle included a melting step (95°C, 10 sec) and a combined annealing and amplification step (60°C, 30 sec). To ensure that the detected fluorescence was the result of a specific amplicon, a melting curve analysis was performed for each run. The fluorescence threshold value was calculated using the Mastercycler ep realplex 1.5 software and the CalQplex algorithm (Eppendorf, Germany). Gene expression levels were determined by applying the ΔΔCt method employing β-Actin as endogenous control.

### Capillary Gel Electrophoresis

Similarly to gel electrophoresis, capillary gel electrophoresis can be used to separate DNA fragments due to their size in an electric field. Thus, capillary gel electrophoresis was applied on generated PCR-products in the course of the qualitative characterization of mRNA-expression for the applied SC lines. To equalize variations in different capillaries an alignment marker was implemented in every run and analysis was performed by the BioCalculator software 1.0. To determine the size of separated DNA fragments a DNA size marker was used (all Qiagen, Germany). Finally, the results were displayed in gel image format.

### Immunocytochemistry

Cells cultivated on glass cover slips for at least 2 days in co-culture with rat brain biopsies were washed in PBS, fixed in methanol: acetone (7∶3) containing 1 µg/ mL DAPI (Roche, Switzerland) for 5 min at 20°C and rinsed three times in PBS. After saturation of non-specific binding by incubation with 1.65% normal goat serum (Vector Laboratories, CA, USA) diluted in PBS for 20 min at room temperature, cells were incubated with primary antibody against α-SMA (1∶100, mouse; DAKO, Denmark), CK18 (1∶800, mouse; Sigma-Aldrich, Germany), Glial Fibrillary Acidic Protein (GFAP, 1∶100, rabbit; DAKO, Denmark), Ki67 (1∶500, rabbit; Novitec, Germany), Nestin (1∶100, mouse; Chemicon, Germany), Neurofilament-mix (NF, 1∶500, rabbit; Serotec, Germany), Oct4 (1∶100, rabbit; Santa Cruz, USA), pan-Cytokeratin (pan-CK, 1∶100, mouse; Sigma-Aldrich, Germany), Sox2 (1∶100, rabbit; Abcam, UK) and Vigilin (1∶200, rabbit; Charli Kruse) diluted in TBST containing 0.1% bovine serum albumin (PAA Laboratories, Austria) in a humid chamber for 60 min at 37°C. After rinsing three times with PBS, cells were incubated with Cy3-labeled anti-mouse IgG (1∶400, Dianova, Germany) and FITC-labeled anti-rabbit IgG (1∶200, Dianova, Germay) in a humid chamber for another 60 min at 37°C. Glass cover slips were washed again three times in PBS, covered in Vectashield mounting medium (Vector Laboratories, CA, USA) and analyzed with a fluorescence microscope (Axioscope 2, Zeiss, Germany). To quantify the staining overall and positive cell numbers from three characteristic images were determined. The length of cell extensions was measured using the length measuring tool of the Zeiss software AxioVision Rel. 4.7.

### Growth Factor Antibody Array

In order to identify growth factors secreted during the co-culturing of SCs with rat brain biopsies a human growth factor antibody array (human growth factor antibody array G Series 8, RayBiotech, USA) was used. This antibody array is also usable for the detection of rat growth factors. Hereby, media supernatants (co-culture, only cells, only brain, only medium/all after two days in culture) from five independently performed experiments with pancreatic SCs were brought onto a glass surface spotted with antibodies against 41 different growths factors. The procedure was done following of the manufacturer's protocols. After blocking nonspecific binding sites on the chip over-night, it was incubated with 100 µL of each supernatant (sterile filtered and containing 1 µL of an internal control) in separate incubation chambers for two hours. After several washing steps, attached growth factors were detected via an additional two-hour-incubation with growth factor-specific biotinylated antibodies followed by over-night incubation with fluorescence-labeled streptavidin. The detection of antibody-coated spots on the chip was done with the Axon GenePix Array Scanner (Molecular Devices, USA) at 532 nm; the results of the five independent experiments were compared and factors showing up in at least four of five cases were considered for the final, semi-quantitative analysis.

## Results

### PSCs, PDSCs and SDSCs Demonstrate Spontaneous Multi-lineage Differentiation Potential

In order to characterize the different populations of PSCs, PDSCs and SDSCs in respect to their stemness and differentiation potential within this system, we investigated specific and well-recognized neuronal SC markers and markers of the three germ layers on the mRNA and protein level.

**Figure 1:**
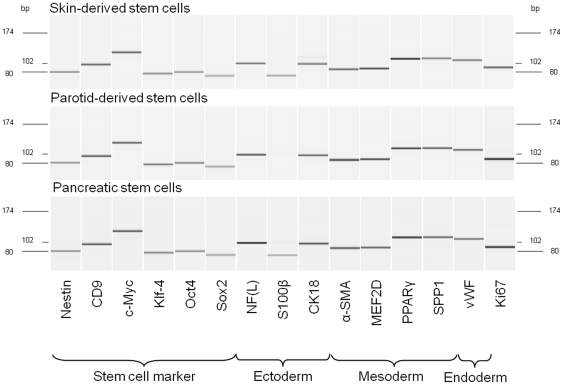
Characterization of mRNA-expression in skin-derived and glandular stem cells. SDSC, PDSC and PSC were analyzed with regard to their transciptome by quantitative RT-PCR and capillary gel electrophoresis. Hereby they were shown to consistently transcribe markers for stemness, proliferation (Ki67) as well as all three germ layers (with exception for S100β in the case of the PDSC).

**Figure 2:**
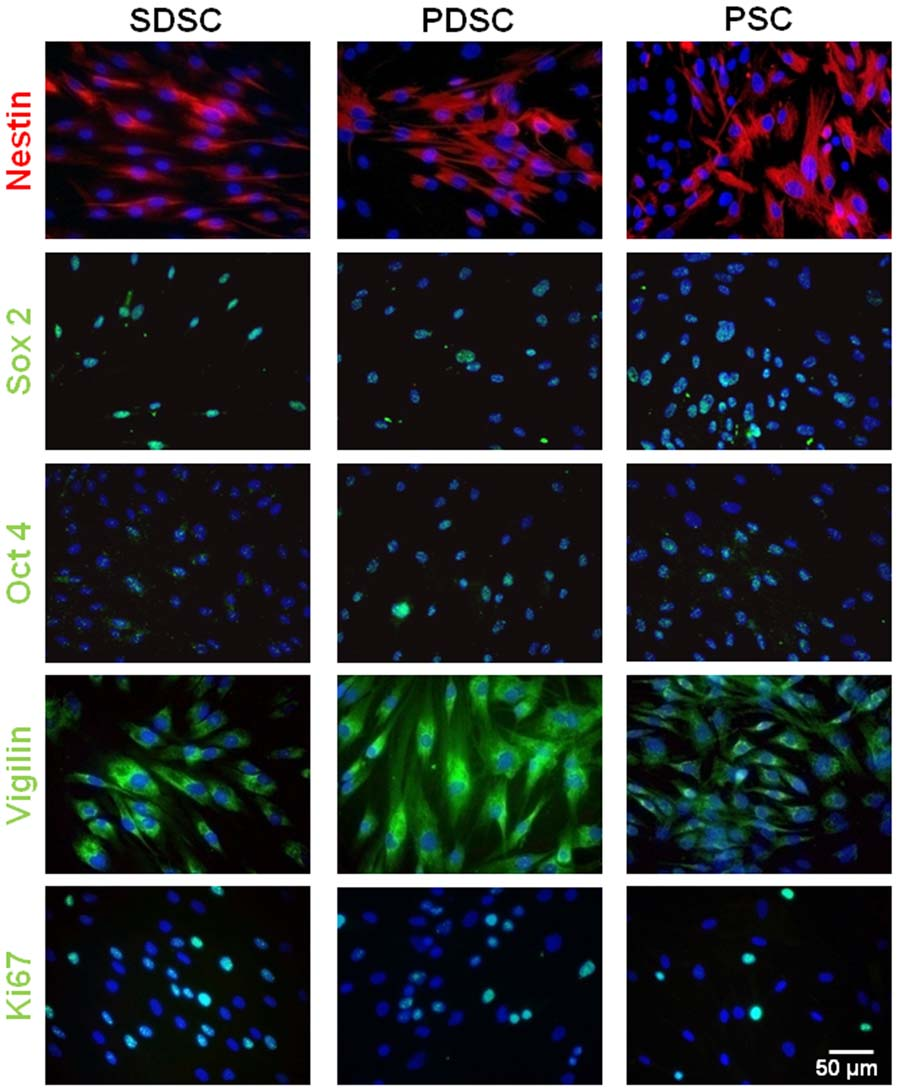
Immunocytochemical characterization of skin- and gland-derived stem cells concerning their stemness. SDSC, PDSC and PSC generally express the stem cell markers Nestin, Sox 2 and Oct 4 as well as marker proteins for protein synthesis (Vigilin) and proliferation (Ki67). Nuclei were counterstained with DAPI (blue).

**Figure 3:**
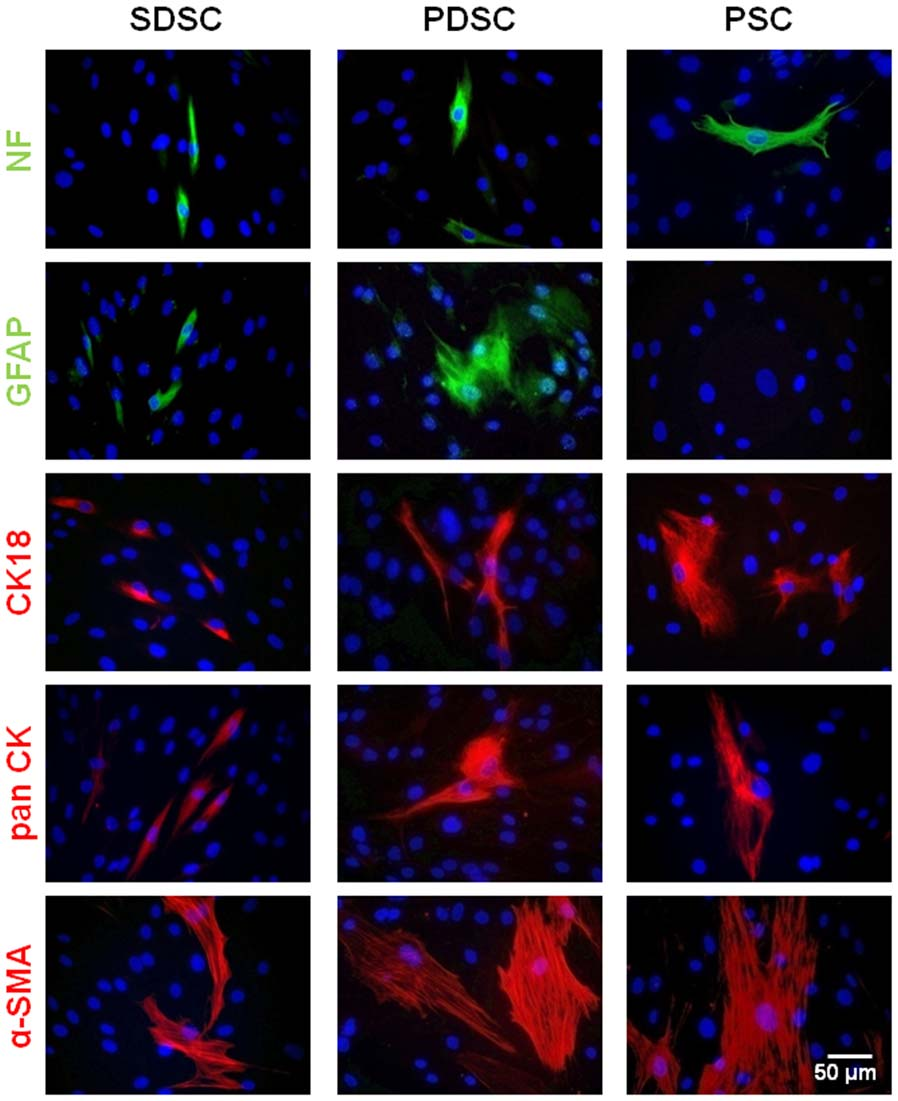
Immunocytochemical characterization of skin- and gland-derived stem cells concerning their differentiation capability. The used stem cell lines (SDSC, PDSC, PSC) spontaneously differentiate *in vitro* and, in the course of this differentiation, express marker proteins for the ectodermal (NF, GFAP, CK18, panCK) and mesodermal (α-SMA) germ layer. Nuclei were counterstained with DAPI (blue).

mRNA expression analysis showed that the SC markers *Nestin*, *CD9*, *c-Myc*, *Klf4*, *Oct4* and *Sox2* could be detected in all three SC populations (Fig. 1). An immunocytochemical staining corroborated the protein expression of Nestin, Sox2 and Oct4 (Fig. 2). Furthermore, the majority of the investigated SCs displayed a positive staining for Vigilin, an ubiquitous RNA-binding protein found in cells with ongoing translational activity (Fig. 2). To show the proliferation activity of the SCs, mRNA and protein expression of the proliferation marker Ki67 was analyzed and demonstrated proliferation in all tested SC types *in vitro* which was in line with the Vigilin expression (Fig. 1, 2). *In vitro* cultivation also displayed high plasticity of the analyzed SC populations as marker proteins of all three germ layers and especially of ectodermal differentiation were detectable. Among them NFs were significantly enriched in SDSCs, PDSCs as well as in PSCs on both the mRNA and protein level (Fig. 1, 3). Whereas GFAP immunoreactivity, a marker for neuroectodermal cells and *S100β-*mRNA, a marker for Schwann cell differentiation, could be only detected in SDSCs and PDSCs (Fig. 1, 3). CK18 was observable at the mRNA (Fig. 1) and protein level (Fig. 3); pan CK, a mixture of different cytokeratin types, was detected on the protein level in all three SC populations (Fig. 3). The mesodermal differentiation marker α-SMA (myogenic) was shown on the mRNA (Fig. 1) and protein level (Fig. 3) in the tested SC populations. Additional markers such as *MEF2D* (myogenic), *PPARγ* (adipogenic) and *SPP1* (osteogenic) were detected at the mRNA level (Fig. 1) in all three populations. Endodermal differentiation was detected by mRNA expression of *vWF* (Fig. 1). These results encouraged us to use these distinct SC populations for testing the influence of our newly established co-culture system on their neuronal differentiation potential.

### Co-culture with Rat Brain Biopsies and Induced Differentiation of PSCs, SDSCs and PDSCs

In order to evaluate the influence of co-cultured rat brain biopsies on the neuronal differentiation of glandular and skin-derived SCs, immunocytochemical stainings and qPCR analysis were performed. Beside the adult SC and neuroectodermal marker Nestin, the expression of various neuronal differentiation markers like NF, β *III Tubulin*, *PGP9.5* and *NSE* was determined. α-SMA served as an indicator for mesodermal differentiation.

**Figure 4:**
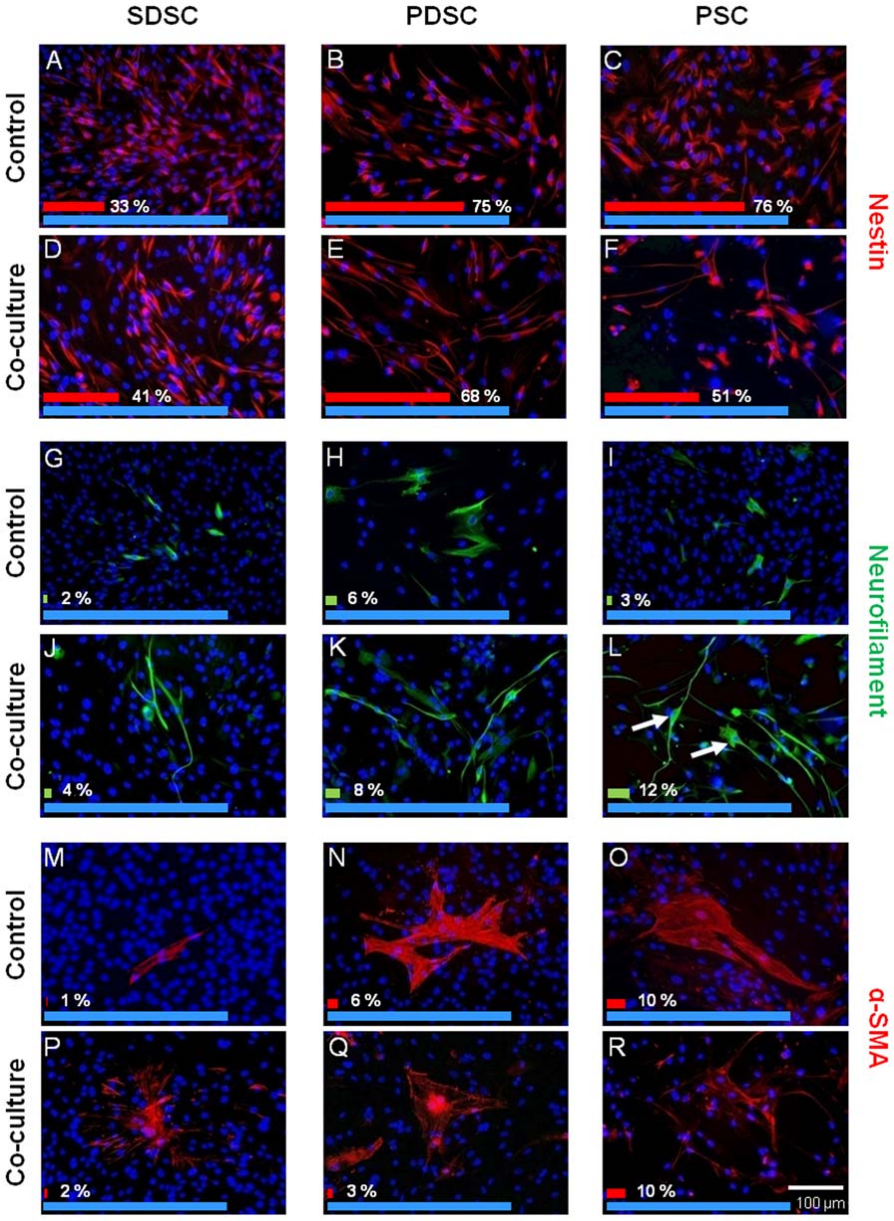
The effect of brain co-culture on the protein expression of skin-derived and glandular stem cells. Co-cultivation with rat brain biopsies led to comparable changes in the expression of some structure proteins in all three human stem cell populations. In the case of Nestin-expressing cells a distinctive decrease by up to one third could be detected, with the slight exception of the SDSC (**A–F**). The percentage of NF-positive cells increased in all populations up to 4-fold, accompanied by the general exhibition of an elongated morphology resembling uni- and bipolar neurons (**G–L**; arrows). α-SMA-positive cells decreased in number and/or showed a visible degradation of the actin filaments (**M–R**). Nuclei were counterstained with DAPI (blue). Blue bars represent the total cell count (via DAPI) in each image, red and green bars the relative amount of positively stained cells.

**Figure 5:**
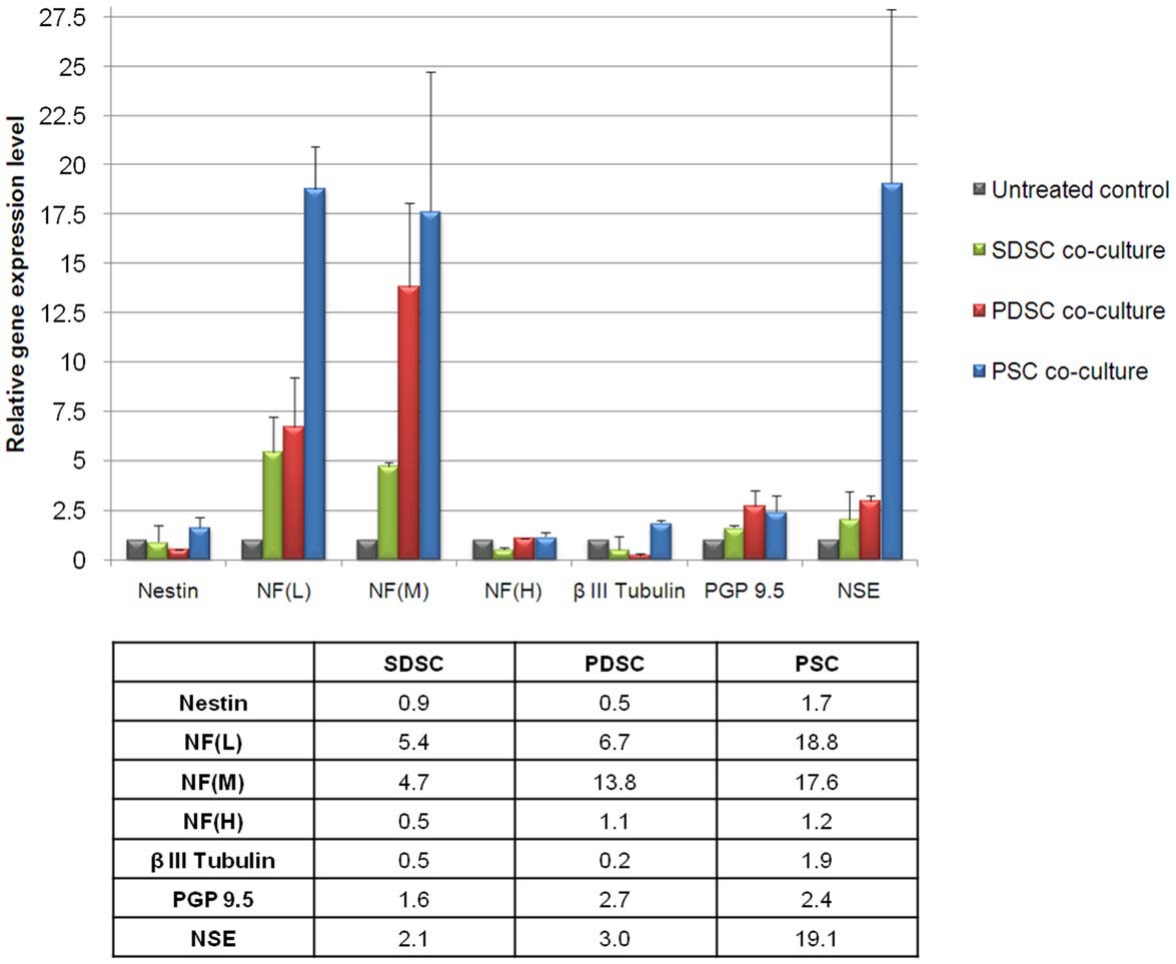
Co-culture-induced alterations in the expression of neural genes by skin-derived and glandular stem cells. The transcriptional activity of brain biopsy co-cultured stem cells was analyzed by quantitative PCR and set in relation to untreated control approaches (representing the value ‘1’). Stimulation resulted in relatively minor variations of the stem cell marker Nestin, whereas the expression of NF(L) and NF(M) was enhanced in all populations and thereby increased up to the factor 18.8 and 17.6 in PSCs, respectively. For the expression of NF(H) almost no alteration was observable. In the case of β III Tubulin some reduction in the mRNA expression level was detectable for SDSC and PDSC; for PSC a slight increase was measurable. The mRNA level of the neuron-specific enzymes PGP9.5 and neuron specific enolase (NSE) was increased after stimulation in all stem cell populations up to the 19.1-fold for NSE in PSC.

**Figure 6:**
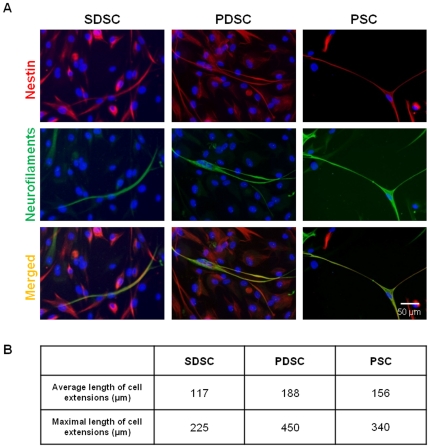
Neuron-like development in skin- and gland-derived stem cells during co-culture. An immunocytochemical staining revealed the co-localization of the neuroprogenitor marker Nestin and the neuronal cell marker Neurofilament in co-culture stimulated SDSC, PDSC and PSC (**A**). Furthermore, the establishment of axon-like cell processes with lengths up to 450 µm could be detected after co-cultivation (**B**).

In populations of SDSCs about 33% of the cells were positive for the intermediate filament Nestin (Fig. 4A). After co-cultivation with rat brain biopsies for the period of two days the total amount of Nestin-expressing cells increased 1.3-fold up to 41% (Fig. 4D), whereby the transcriptional activity for this protein remained unchanged (Fig. 5). In the control approach about 2% of the cells expressed NF and featured a small spindle-shaped cellular body (Fig. 4G). Co-cultivation with rat brain biopsies led to a change in the morphological appearance as the NF-positive cells became elongated with axon-like processes up to 225 µm (Fig. 6B); the occurrence of those cells was 4% (Fig. 4D). This increase could also be measured on the mRNA level. The relative gene expression level of *NF(L)* and *NF(M)* was 5.4 and 4.7 times higher, respectively, whereas the expression of *NF(H)* diminished by half. In addition, it could be shown that cells were double immunoreactive for Nestin and NF (Fig. 6A). For *β III Tubulin*, qPCR analysis revealed a 0.5-fold decrease while *PGP9.5* and *NSE* increased 1.6-fold and 2.1-fold respectively (Fig. 5). The number of α-SMA-positive cells revealed a slight increase from 1% in untreated populations (Fig. 4M) to 2% after co-culture (Fig. 4P). Nevertheless, the co-cultivation led to an instable and disaggregating actin skeleton as indicated in figure 4P.

In the unstimulated PDSCs 75% of the cells were Nestin-positive (Fig. 4B). Incubation with rat brain biopsies caused a marginal decrease in Nestin expression down to 68% and a morphological alteration as the cells became elongated with characteristical neuron-like extensions (Fig. 4E). Concomitantly the amount of NF-positive cells rose slightly from 6% up to 8% (Fig. 4H/K). These cells also exhibited an elongated morphology with cell extensions up to 450 µm (Fig. 6B). It could be shown at the protein level that some of these cells were double immunoreactive for Nestin and NF (Fig. 6A). Additionally, a significant increase could be detected on the relative mRNA expression level of *NF(L)* and *NF(M), being* 6.7 and 13.8 times higher subsequent to co-culture experiments. For *NF(H)* no changes in the expression levels were measurable. The mRNA-level for *β III Tubulin* decreased up to 0.2 times. In contrast the mRNA expression of other neuronal markers like *PGP9.5* and *NSE* increased 2.7-fold and 3-fold respectively (Fig. 5). Finally, an immunocytochemical analysis of α-SMA revealed a decrease of positive cells from 6% down to 3% in co-culture stimulated PDSCs (Fig. 4N/Q) with a concomitant degradation of the actin skeleton (Fig. 4Q), demonstrating inhibition of mesodermal differentiation within this population.

The highest amount of Nestin-positive cells was detected in PSC populations. In the control set 76% of the PSCs were positively stained whereby those cells exhibited a flattened morphology (Fig. 4C). The co-cultivation with rat brain tissue led to the decrease of Nestin-positive cells down to 50% and an elongated cell shape (Fig. 4F). Hereby, the PSC population demonstrated the strongest effect of the rat brain biopsies on neuronal differentiation as the total amount of NF-positive cells increased from 3% up to 12% (Fig. 4I/L). Moreover, a distinct morphological change associated with the appearance of up to 340 µm long uni- and bipolar axon-like structures was observed for NF-positive cells (Fig. 4L, arrows; Fig. 6B). Furthermore, it was possible to detect some cells immunoreactive for Nestin as well as NF (Fig. 6A). qPCR corroborated the results of the immunocytochemical analysis; the relative gene expression of *NF(L)* and *NF(M)* was enhanced 18.8-fold and 17.6-fold, respectively. Furthermore, a doubling in the expression of *β III Tubulin* and *PGP9.5* was detected and *NSE* was expressed even 19-times higher (Fig. 5). Our co-cultivation model had almost no effect on the amount of α-SMA-positive cells though the actin skeleton was in a degradation process in all of these cells (Fig. 4O/R).

### Determination of Secreted Growth Factors in the Co-culture System

**Figure 7:**
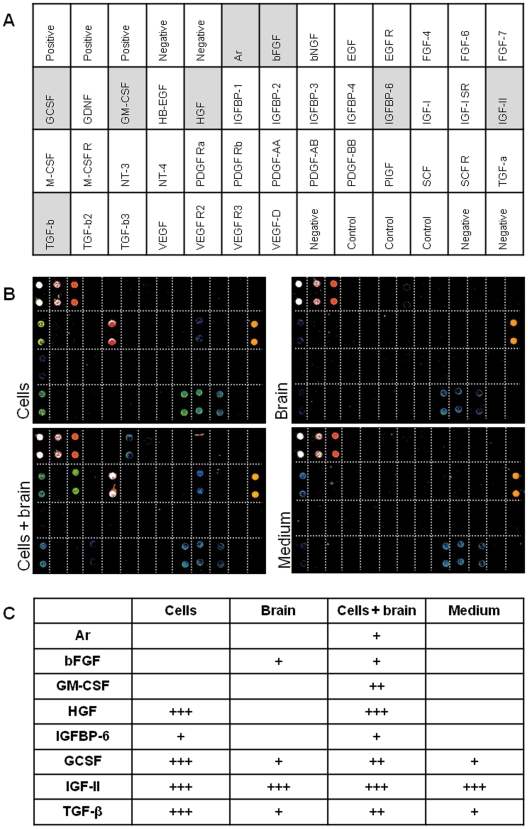
Detection of secreted growth factors via microarray. Supernatants of co-cultures and controls were tested for the presence of growth factors via a microarray-based technique (**A**). The processed arrays were semi-quantitatively analyzed by fluorescence, whereby representative images for every approach are shown (**B**). Growth factors that were found in at least four of five experiments were comparatively described in their expression based on the fluorescence signals (blue = weak/+; green-yellow = moderate/++; red-white = strong/+++) (**C**).

Media supernatants of co-culture experiments as well as separately cultured SC populations and isolated brain biopsies were tested for 41 growth factors via a microarray-based technique (Fig. 7). This was done in order to identify factors that might be responsible for the enhanced neuronal differentiation of PSCs during co-culturing. We detected that PSCs themselves strongly express Hepatocyte Growth Factor (HGF), as well as lower amounts of the Insulin-like Growth Factor Binding Protein 6 (IGFBP-6). For the employed rat brain biopsies lower secretion of basic Fibroblast Growth Factor (bFGF) was detected. The combination of adult human SCs and rat brain biopsies during the co-culture led to a distinct variation in the growth factor composition of the analyzed supernatants compared to the single approaches. Besides the additional presence of Amphiregulin (Ar) a moderate secretion of Granulocyte Macrophage Colony-stimulating Factor (GM-CSF) was found in the co-culture approaches. Due to the use of fetal bovine serum in the culture medium several growth factors (Granulocyte Colony Stimulating Factor (GCSF), Transforming Growth Factor beta (TGF-β) and Insulin-like Growth Factor II (IGF-II) were already present in the untreated supernatants.

## Discussion

In this work we propose a xenogeneic co-culture approach of rat brain biopsies and adult human SCs to evaluate the neuronal differentiation potential of SCs. The presented system is an effective tool to predict the behavior of SCs after transplantation into a neuronal environment by mimicking the situation *in vivo*. This is important since the potential treatment of neurodegenerative disorders by the application of autologous SCs comprises the guided neuronal differentiation of these cells *in vivo* as a prerequisite.

To demonstrate the applicability of this novel system, we used different adult SC populations derived from human parotid gland, pancreas and skin, which were shown to possess a high multi-lineage plasticity. Prior to the co-culture experiments we pointed out the similarity of glandular and skin-derived SCs in regard to the expression of various SC and differentiation markers as was described for adult human SCs originated in glandular tissues and skin before [19], [22].

Nestin, an adult SC marker [23] but also a marker for neuroectodermal progenitor cells [24] could be detected in different quantities in the studied SC populations. The co-culture approach with rat brain tissue showed that the cell population with the highest basal Nestin expression (PSC, 76%) led to the highest yield of NF-positive cells after co-culture while in skin-derived SCs with only 33% Nestin-positive cells the co-culture approach resulted in the lowest efficiency of neuronal-like cells, even though an increase in neuronal differentiated cells was also obvious. Furthermore, indicated by the detection of double-immuno-positive ‘transition state’ cells, a differentiation of NF-positive cells from Nestin-expressing cells could be demonstrated, causing a decrease of Nestin-positive cells in the course of the co-culture. Referring to the development of neurons [25] it could also be shown that the expression of early NFs (light and medium) was upregulated. In particular NF(M), which appears with the beginning of neurite formation, was highly upregulated in PSC and PDSC where the longest cellular extensions were detectable. The expression of late NF(H), which occurs after neuronal maturation, was not affected by co-culture. This could be due to the short incubation time of 48 hours which did not allow the cells to completely differentiate into a neuronal cell type. Likewise the expression of β III Tubulin, which is prominently expressed during fetal and postnatal development of the human nervous system [26], was not enhanced by neural tissue stimulation. However, in addition to a shift in the expression of neuron-associated intermediate filaments from Nestin- to NF-expressing cells with a concomitant alteration of the cell morphology towards a neuron-like phenotype, an enhanced expression of neuron specific enzymes could be observed, corroborating the presumption of a partial differentiation along the neuronal cell lineage. In addition, a significant depletion of α-SMA indicates inhibition of mesodermal differentiation and therefore provides further evidence for a guided differentiation into the neuronal lineage.

Since SCs and brain biopsies were spatially divided only by a porous membrane, sharing the same cultivation medium, one can assume that soluble factors released from the rat brain effected the SC differentiation. Anwar et al. [15] proposed that factors secreted especially by necrotic nerve tissue segments might induce a neuronal differentiation in adult SCs. This could also be the case in our co-culture approach, in which the SCs share the same medium as the cut and thus ‘severely injured’ and partially necrotic brain biopsies. Mutual interaction between the SCs and the brain biopsies by secretion of soluble factors, and autocrine effects of such factors on SCs and biopsies is likely a feature of our co-culture system. We therefore tested the media supernatants on factors corresponding to those factors and detected bFGF, a brain injury-related factor with neurogenic properties [27] in the brain-conditioned samples. Additionally, with Amphiregulin [28] and GM-CSF [29] two other factors of that category were found in the supernatants of the co-cultures. Since these factors were not present in the separately cultured brain biopsies and SCs the assumption of an interaction between SCs and brain biopsies could be suggested, which led to the secretion of these factors. In this context it should be noted that expression of Amphiregulin by neuronally differentiated cells of mesenchymal origin has been shown previously [30]. So, there might be the possibility that this factor was secreted by the differentiating SCs. The expression of several factors like HGF and IGFBP-6 by the pancreatic SCs is actually not surprising and had to be expected, taking in consideration the wide variety of growth factors generally expressed by adult SCs [31]. Factors which were already present in the cultivation medium have not been included in the analysis, since treated and untreated SC could have been influenced by these factors likewise. Astrocytes are the main cell type in the rat brain, so that factors secreted by these cells in particular may be responsible for the induction of neuronal differentiation in the tested human SC populations. One possible factor which is known to be expressed by astrocytes is the Glial Cell-derived Neurotrophic Factor (GDNF) [32]. Following brain injury GDNF is upregulated [33]; this could also be the case in our co-culture system assuming that cells in the dissected brain tissue behave like their counterparts *in vivo*. No GDNF was detected in our co-culture supernatants, which could be due to the insufficient sensitivity of the used assay. Nevertheless, Suarez-Rodriguez et al. [34] showed that there are Nestin-positive cells which express the Glial-derived Neurotrophic Factor Receptors (GFR)alpha-1, GFRalpha-2, and GFRalpha-3.

At that point it has to be underlined that we here propose a co-culture system which works across different species as the used SCs were from human origin and the brain biopsies as well as the soluble factors influencing the SCs were obtained from rats. Such a xenogeneic approach is not uncommon as Anwar et al. [10] already described the differentiation of human neural SCs into dopaminergic neurons by co-culture with rat brain slices. Many growth factors are evolutionarily highly conserved [35], [36] and the cross-reactivity of growth factors between different species plays a decisive role while using fetal bovine serum (FBS) in the routine cultivation of any cell type [37], [38], [39]. Furthermore, an analysis of the amino acid sequences of potentially important growth factors via BLAST (http://blast.ncbi.nlm.nih.gov) reveals partial or high homologies between human and rat, so that one can principally assume a sufficient interaction of the rat brain-derived growth factors with receptors on the human SCs as well as with the antibodies on the human growth factor array.

Guided differentiation of human SCs into neuronal cells by the means of co-culture with brain biopsies can be a promising model to predict the *in vivo*-behavior of transplanted SCs in the course of neuroregenerative cell-based therapies. Because of accessibility of adult parotid and skin-derived SCs such cells may constitute a promising source for autologous cell transplantation and makes it therefore necessary to further study the properties of such cells in a given context. For example the suitability to integrate into the brain tissue has to be explored on brain slices and in *in vivo*-studies. In addition, test systems for neuronal drug discovery based on adult SC-derived and predifferentiated neuronal cells may be a conceivable tool for the pharmaceutical industry. Hereby our co-culture system provides a first step to identify suitable factors for the neuronal differentiation of adult human SCs, which will be investigated in future experiments. Furthermore, experiments should be conducted in order to investigate the functionality and types of generated neurons.

## References

[1] Perrier AL, Tabar V, Barberi T, Rubio ME, Bruses J (2004). Derivation of midbrain dopamine neurons from human embryonic stem cells.. Proc Natl Acad Sci U S A.

[2] Schuldiner M, Eiges R, Eden A, Yanuka O, Itskovitz-Eldor J (2001). Induced neuronal differentiation of human embryonic stem cells.. Brain Res.

[3] Lee G, Chamber SM, Tomishima MJ, Studer L (2010). Derivation of neural crest cells from human pluripotent stem cells.. Nat Protoc.

[4] Drukker M, Katz G, Urbach A, Schuldiner M, Markel G (2002). Characterization of the expression of mhc proteins in human embryonic stem cells.. Proc Natl Acad Sci U S A.

[5] McLaren A (2001). Ethical and social considerations of stem cell research.. Nature.

[6] Li W, Yamashita H, Hattori F, Chen H, Tohyama S (2011). Simple autogeneic feeder cell preparation for pluripotent stem cells.. Stem Cell Res.

[7] Blum B, Benvenisty N (2008). The tumorigenicity of human embryonic stem cells.. Adv Cancer Res.

[8] Sanchez-Ramos J, Song S, Cardozo-Pelaez F, Hazzi C, Stedeford T (2000). Adult bone marrow stromal cells differentiate into neural cells in vitro.. Exp Neurol.

[9] Alexanian AR (2010). An efficient method for generation of neural-like cells from adult human bone marrow-derived mesenchymal stem cells.. Regen Med.

[10] Khoo ML, Tao H, Meedeniya AC, Mackay-Sim A, Ma DD (2011). Transplantation of neuronal-primed human bone marrow mesenchymal stem cells in hemiparkinsonian rodents.. PLoS One.

[11] Cho KJ, Trzaska KA, Greco SJ, McArdle J, Wang FS (2005). Neurons derived from human mesenchymal stem cells show synaptic transmission and can be induced to produce the neurotransmitter substance p by interleukin-1 alpha.. Stem Cells.

[12] Prabhakaran MP, Venugopal JR, Ramakrishna S (2009). Mesenchymal stem cell differentiation to neuronal cells on electrospun nanofibrous substrates for nerve tissue engineering.. Biomaterials.

[13] Scintu F, Reali C, Pillai R, Badiali M, Sanna MA (2006). Differentiation of human bone marrow stem cells into cells with a neural phenotype: diverse effects of two specific treatments.. BMC Neurosci.

[14] Tao H, Rao R, Ma DDF (2005). Cytokine-induced stable neuronal differentiation of human bone marrow mesenchymal stem cells in a serum/feeder cell-free condition.. Dev Growth Differ.

[15] Anwar MR, Andreasen CM, Lippert SK, Zimmer J, Martinez-Serrano A (2008). Dopaminergic differentiation of human neural stem cells mediated by co-cultured rat striatal brain slices.. J Neurochem.

[16] Kruse C, Birth M, Rohwedel J (2004). Pluripotency of adult stem cells derived from human and rat pancreas.. Applied Physics A.

[17] Rotter N, Oder J, Schlenke P, Lindner U, Böhrnsen F (2008). Isolation and characterization of adult stem cells from human salivary glands.. Stem Cells Dev.

[18] Petschnik AE, Klatte JE, Evers LH, Kruse C, Paus R (2010). Phenotypic indications that human sweat glands are a rich source of nestin-positive stem cell populations.. Br J Dermatol.

[19] Kajahn J, Gorjup E, Tiede S, von Briesen H, Paus R (2008). Skin-derived human adult stem cells surprisingly share many features with human pancreatic stem cells.. Eur J Cell Biol.

[20] Egaña JT, Danner S, Kremer M, Rapoport DH, Lohmeyer JA (2009). The use of glandular-derived stem cells to improve vascularization in scaffold-mediated dermal regeneration.. Biomaterials.

[21] Kruse C, Kajahn J, Petschnik AE, Maass A, Klink E (2006). Adult pancreatic stem/progenitor cells spontaneously differentiate in vitro into multiple cell lineages and form teratoma-like structures.. Ann Anat.

[22] Gorjup E, Danner S, Rotter N, Habermann J, Brassat U (2009). Glandular tissue from human pancreas and salivary gland yields similar stem cell populations.. Eur J Cell Biol.

[23] Wiese C, Rolletschek A, Kania G, Blyszczuk P, Tarasov KV (2004). Nestin expression–a property of multi-lineage progenitor cells?. Cell Mol Life Sci.

[24] Yaworsky PJ, Kappen C (1999). Heterogeneity of neural progenitor cells revealed by enhancers in the nestin gene.. Dev Biol.

[25] Carden MJ, Trojanowski JQ, Schlaepfer WW, Lee VM (1987). Two-stage expression of neurofilament polypeptides during rat neurogenesis with early establishment of adult phosphorylation patterns.. J Neurosci.

[26] Katsetos CD, Herman MM, Mörk SJ (2003). Class iii beta-tubulin in human development and cancer.. Cell Motil Cytoskeleton.

[27] Komobuchi H, Hato N, Teraoka M, Wakisaka H, Takahashi H (2010). Basic fibroblast growth factor combined with biodegradable hydrogel promotes healing of facial nerve after compression injury: an experimental study.. Acta Otolaryngol.

[28] Nilsson A, Moller K, Dahlin L, Lundborg G, Kanje M (2005). Early changes in gene expression in the dorsal root ganglia after transection of the sciatic nerve; effects of amphiregulin and pai-1 on regeneration.. Brain Res Mol Brain Res.

[29] Hayashi K, Ohta S, Kawakami Y, Toda M (2009). Activation of dendritic-like cells and neural stem/progenitor cells in injured spinal cord by gm-csf.. Neurosci Res.

[30] Tondreau T, Dejeneffe M, Meuleman N, Stamatopoulos B, Delforge A (2008). Gene expression pattern of functional neuronal cells derived from human bone marrow mesenchymal stromal cells.. BMC Genomics.

[31] Ramalho-Santos M, Yoon S, Matsuzaki Y, Mulligan RC, Melton DA (2002). "stemness": transcriptional profiling of embryonic and adult stem cells.. Science.

[32] Schaar DG, Sieber BA, Dreyfus CF, Black IB (1993). Regional and cell-specific expression of GDNF in rat brain.. Exp Neurol.

[33] Cheng Q, Liberto VD, Caniglia G, Mudò G (2008). Time-course of gdnf and its receptor expression after brain injury in the rat.. Neurosci Lett.

[34] Suárez-Rodríguez R, Belkind-Gerson J (2004). Cultured nestin-positive cells from postnatal mouse small bowel differentiate ex vivo into neurons, glia, and smooth muscle.. Stem Cells.

[35] Hallböök F, Ibáñez CF, Persson H (1991). Evolutionary studies of the nerve growth factor family reveal a novel member abundantly expressed in xenopus ovary.. Neuron.

[36] Ornitz DM, Itoh N (2001). Fibroblast growth factors.. Genome Biol.

[37] Gimbrone MA, Cotran RS, Folkman J (1974). Human vascular endothelial cells in culture. Growth and DNA synthesis.. J Cell Biol.

[38] Evans MJ, Kaufman MH (1981). Establishment in culture of pluripotential cells from mouse embryos.. Nature.

[39] Ciba P, Schicktanz S, Anders E, Siegl E, Stielow A (2008). Long-term culture of a cell population from siberian sturgeon (acipenser baerii) head kidney.. Fish Physiol Biochem.

